# An Analysis of Maxillary Anterior Teeth Crown Width-Height Ratios: A Photographic, Three-Dimensional, and Standardized Plaster Model's Study

**DOI:** 10.1155/2022/4695193

**Published:** 2022-02-07

**Authors:** Naseer Ahmed, Mohamad Syahrizal Halim, Ayesha Aslam, Zuryati Ab Ghani, Jawad Safdar, Mohammad Khursheed Alam

**Affiliations:** ^1^Prosthodontics Unit, School of Dental Sciences, Health Campus, Universiti Sains Malaysia, Kubang Kerian, 16150 Kota Bharu, Kelantan, Malaysia; ^2^Department of Prosthodontics, Altamash Institute of Dental Medicine, Karachi 75500, Pakistan; ^3^Conservative Dentistry Unit, School of Dental Sciences, Health Campus, Universiti Sains Malaysia, Kubang Kerian, 16150 Kota Bharu, Kelantan, Malaysia; ^4^Department of Prosthodontics, Army Medical College/Armed Forces Institute of Dentistry, National University of Medical Sciences, Islamabad, Pakistan; ^5^Prosthodontics Unit, School of Dental Sciences, Health Campus, Universiti Sains, Kubang Kerian, 16150 Kota Bharu, Kelantan, Malaysia; ^6^Department of Oral and Maxillofacial Surgery. Dow Dental College, Dow University of Health Sciences, Karachi, Pakistan; ^7^Department of Preventive Dentistry, College of Dentistry, Jouf University, Sakaka, Al Jouf 72345, Saudi Arabia; ^8^Centre for Transdisciplinary Research (CFTR), Saveetha Dental College, Saveetha Institute of Medical and Technical Sciences, Saveetha University, Chennai 600077, India; ^9^Department of Public Health, Faculty of Allied Health Sciences, Daffodil International University, Dhaka 1207, Bangladesh

## Abstract

**Objective:**

To analyze the width and height ratios of maxillary anterior teeth at different crown levels through photographs, 3D, and plaster dental model techniques in a subset of the Pakistani population. *Material and Methods*. This clinical study consisted of 230 participants. The maxillary impression, standardized photographs, and models were constructed for crown width and height analysis. The SPSS version 25 was used for statistical analysis. Descriptive statistics were carried out for mean, standard deviation, and percentage calculation of teeth width and height, gender, and age of participants. Paired *t*-test analysis was carried out to compare the dependent variables (teeth size, width, and height ratios) with independent variables (techniques applied, side disparity). A *p* value of ≤ 0.05 was considered statistically significant.

**Results:**

The mean width and height of maxillary anterior teeth obtained through photographs, 3D, and plater models were statistically different. The 3D dental model analysis showed reliable and accurate results. The mean width and height ratio of teeth were different on both sides of the arch. There was a significant difference (*p* = 0.001) in crown width-height ratios at different crown levels.

**Conclusion:**

The width and height ratios in the studied population were different at various crown levels. The dimensions of teeth varied from the incisal to the cervical part of the crown. Hence, rather than relying on a single, fixed ratio of 78% to 80% suggested by researchers for anterior teeth, the clinician should adopt different crown width-height ratios to restore teeth with the optimum esthetic outcome.

## 1. Introduction

Esthetic restoration of smiles is a complicated process mandating a multidisciplinary approach [1]. The major concern for patients seeking esthetic dental treatment is the appearance of anterior teeth [2]. Among other parameters, the dimensions of maxillary anterior teeth are the most significant factors in achieving harmonious and esthetic outcomes [3]. However, defining ideal tooth dimensions is rather difficult owing to individual variations and proximal tooth wear [4].

Maxillary anterior teeth, being the most prominent ones, are paramount to the restoration of anterior dental esthetics as well as overall facial esthetics [5]. Selection of appropriate crown length and width is essential to creating esthetically pleasing smiles. Crown width to length ratio is considered as the most stable parameter, essential to achieve a harmony between dental esthetics and facial contours [6]. For maxillary anterior teeth, several theories exist suggesting the ideal proportions that may result in esthetic results such as golden proportion, golden percentage, and recurring esthetic dental proportion [7].

The ratio between height and width of maxillary anterior has been assessed using several methods. Sterret et al. [8] employed dental casts to assess the crown width to height ratio (CWHR) of maxillary anterior teeth. They reported a CWHR of 85%, 76%, and 77% for males and 86%, 79%, and 81% for females for maxillary central incisors, lateral incisors, and canines, respectively. Magne et al., in contrast, utilized extracted teeth to measure CWHR and observed mean ratios of 78% for central incisors and 73% each for lateral incisors and canines [9]. While Chu [10] reported a CWHR of 78% for all maxillary anterior teeth using “Chu's esthetic proportion gauge,” Shahid et al. [11] and Yuan et al. [5] utilized digital calipers to measure the dimensions of maxillary teeth and reported significant differences only in terms of crown widths and height between males and females and not in CWHR. With advancements in digital dentistry, 3D digital models and software are now being used for easier and faster measurements of dental clinical parameters [7, 12].

As previously stated, knowledge about crown width and height (*W*/*H*) ratio is critical for optimal restoration of maxillary anterior teeth. A study presenting crown width-height ratio of maxillary anterior teeth at different crown levels is lacking. The present study, therefore, is aimed at determining the crown width-height ratio of maxillary anterior teeth at different clinical crown levels utilizing 2D photographs, plaster, and 3D digital dental models. Specific objectives of the study included the following:

- To evaluate the mean mesiodistal widths and incisocervical lengths of maxillary anterior teeth using 2D photographs, 3D models, and standard plaster models
- To evaluate the mesiodistal widths and incisocervical lengths of maxillary anterior teeth using 2D photographs, 3D models, and standard plaster models at different crown levels
- To evaluate the width-height ratios of maxillary anterior teeth at different crown levels
- To compare the mean width-height ratios of maxillary anterior teeth at different crown levels

## 2. Materials and Methods

### 2.1. Study Setting and Sample Size

**Figure 1:**
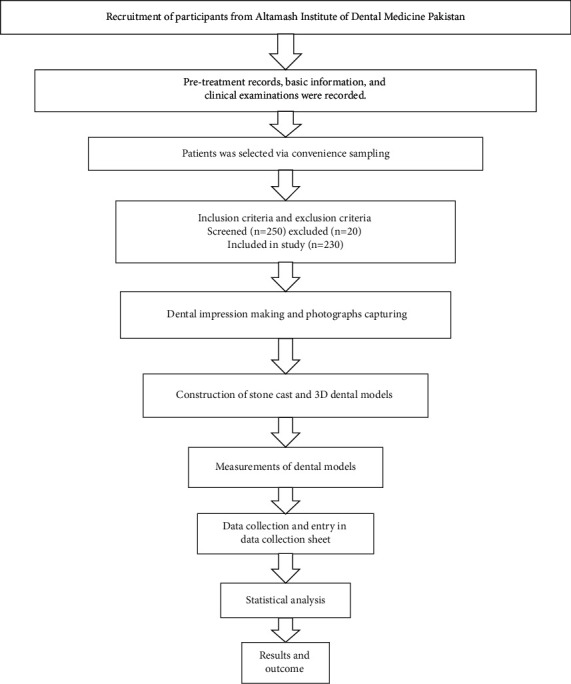
Flow diagram of the study methodology.

This analytical study was carried out at the Altamash Institute of Dental Medicine, Pakistan. A total of 230 subjects participated in this study. The age range of participants was 18 to 30 years. A nonprobability convenience sampling technique was used to recruit participants in this study. The flow diagram of the stepwise methodological approach adopted in this study is described in Figure 1. The sample size was calculated with public service of creative research systems survey software (creative research systems, version 9, Petaluma, California, United States). Considering the width and height ratio of 85.55% for central incisor [13], the estimated sample size at 5% margin of error with 95% confidence interval, 230 individuals with intact natural maxillary anterior teeth were invited to participate in this study, considering the 10,000,000 population.

### 2.2. Participant Recruitment and Ethical Consideration

The ethical permission was obtained from the ethical review board of AIDM number AIDM/EC/06/2019/06 and Universiti Sains Malaysia number USM/JEPeM/19060380. The participants were interviewed. The informed consent for voluntary participation and refusal at any time from the study was carried out for each participant. The form number, nationality, age, gender, height, weight of participants, and contact details were noted. The intraoral and extraoral examination was carried out to eliminate facial malformation, asymmetry, deviation of temporomandibular joint, and difficulty in mouth opening. The participants were also screened for dental caries, any restoration in anterior teeth, malalignment of teeth, gingival inflammation, and history of orthodontic treatment. Two hundred and fifty participants were initially screened to be included in the study. Later, 20 participants were excluded based on malalignment of teeth, facial asymmetry, restored teeth, i.e., composite restoration, crown, and bridgework, subjects with blur/unclear photographs, impression making errors, and broken or destroyed dental casts in the process of fabrication.

### 2.3. Capturing Retracted Smile Intraoral Photograph

**Figure 2:**
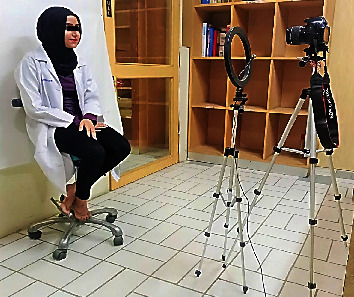
Pictorial illustration showing methodology of obtaining standard digital images with subject in natural head position.

A digital camera (Canon EOS, DSLR Camera, CMOS, 18 MP,1920 X 1080 p/30 fps) was used to capture crisp clear images. The camera was equipped with a built-in magnification lens of (18–55 mm + 75–300 mm) to capture reproducible images. The 1 : 1 macro setting was used for close-up photography of teeth and generally included 4 four maxillary incisors and canine teeth on the sensor. The 1 : 1 setting was used for capturing anterior teeth images with a focus set on subject's central incisor tooth. The camera was set at the 12 o'clock position, mounted on a tripod with a standardized focus and distance of 1.5 meters from the participants to ensure distortion-free images. The surrounding lighting remained the same for all the photographs. A ring flashlight source system (LED-FD,480II, Medike Photo and Video Co., Ltd. Yidoblo, Guangdong, China) was used, and its configuration consisted of a light unit that was mounted next to the camera lens. The design was of a movable type which consisted of a light (fluorescent) that was mounted further from the lens placed in variable custom positions around a circular mounting bracket. A photograph of anterior teeth for assessment of study subjects was taken from the front, with the subject in a seated position. The head position was guided by the investigator to assist the participants in assuming their natural head position. The height of the lens of the camera was adjusted on the tripod to match the level of the incisors for retracted smile image capture. The participants were seated upright with shoulders and head held straight and facing forward, looking straight ahead at the lens of the camera; the natural head position was standardized along both horizontal and vertical axes. In all intraoral photos, the upper and lower lips were retracted to display the maxillary anterior teeth. This procedure was like the protocol described by Bidra et al. [14] (Figure 2).

### 2.4. Maxillary Impression and Dental Cast Making

For the fabrication of the maxillary cast, the perforated type of stainless-steel maxillary impression tray was carefully selected; the tray must cover the hamular notches and fovea palatine and also provide adequate space for 3-4 millimeters for the impression material uniformly. The borders of the tray were extended up to the functional sulcus depth without causing physical discomfort to the participants.

The impressions of the maxillary arch were made of all subjects using irreversible hydrocolloid material (fast setting alginate hydrogum; Zharmack Spa). It was manipulated according to manufacturer's instructions. The errorless impressions after making were washed under running tap water for 10 seconds to remove debris and salivary pellicle. Every impression was immersed in dimethyl ammonium chloride solution (BODE) for 10 minutes to achieve disinfection. After disinfection, a serial number was allotted to each impression for identification purposes. The impressions were poured with type IV dental stone (ISO Type 3, Elite Rock Zharmack Spa). The dental cast was removed from the impression after 30 minutes to avoid errors like dimensional changes and desiccation of the cast by the set impression material. After removal, the cast was coded with a serial number of subjects using a permanent marker. The bases of casts were made with soft plaster using standardized base formers. To obtain a 3D model, the cast was scanned by UP3D Dental Laboratory Scanner (UP360+, 300 × 300 × 400 mm, 3D scanner, Shenzhen, China). The scanner was equipped with 2.0 MP cameras that can scan with high precision up to 6 *μ*m. The full-arch 3D scan was obtained in 20 seconds. The scanned images were displayed on a compatible dental design software (UPCAD, UP3D, Shenzhen, China), then transferred via USB to store in a personal computer.

### 2.5. Plaster, Photographic, and 3D Dental Cast Teeth Width Measurement

The photographic and 3D dental cast mesiodistal width of the maxillary anterior teeth was recorded with a measuring tool in millimeters setting through Photoshop software (Adobe, version 21.0.2, San Jose, California, United States). The plaster dental cast widths were calculated with a sharp-tipped digital Vernier calliper (Neiko 01407A Electronic Digital Calliper), read to the nearest 0.02 mm. The mesiodistal widths of central incisors, lateral incisors, and canines were measured from the facial side.

**Figure 3:**
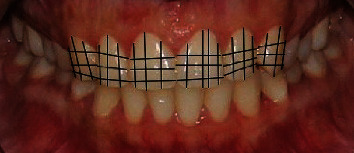
Demonstrating the method applied to measure maxillary anterior teeth width and height.

The width of the maxillary anterior teeth crown was measured at the incisal third, middle third, and cervical third of the crown from the labial aspect. The height of the crown was measured from 3 aspects; the mesial third of the crown from incisal edge to base of interdental papilla similarly at the distal third the crown length was measured in millimeters and recorded. The crown height was also measured at the middle one-third from the incisal edge to the deepest point in the cervical third of the crown (Figure 3). The information regarding teeth measurements obtained from all three sources was recorded and transferred to a computer spreadsheet.

### 2.6. Calculation of Crown Width and Height Ratios

To calculate the crown width and height (*W*/*H*) ratios, the incisal third width was divided by mesial third height, middle third width was divided by middle third crown height, and cervical third width was divided by distal third crown height. This way, the width and height ratio of each tooth was calculated at three different crown levels.

### 2.7. Validity and Reliability Assessment

The data methods and collection were performed by a single operator (N.A.). Initially, for interoperator assessment, the data collection was performed by a senior colleague (J.S.). The data was then subjected to correlation analysis; a strong correlation value of (0.739) was noted between the operator measurements.

To minimize intraoperator errors, each measurement was performed thrice; a constant or mean value of variables was then noted in proforma. Furthermore, 20% of photographs and dental models were assessed after 2 weeks by the same operator. The data was analyzed later by the Dahlberg formula to detect intraoperator reliability through correlation statistics.

For validity purposes, 20% of dental cast measurements that were carried out with a Vernier calliper were compared with 3D dental cast measurements through adobe photoshop software (Adobe, version 21.0.2, San Jose, California, United States). The intraclass correlation coefficient test (ICC) was carried to obtain an association between the two sets of measurements. A strong correlation value of (0.816) was found because of the analysis.

To minimize the photographic error, the actual width of maxillary anterior teeth obtained from the dental cast was divided by perceived width from photographs to obtain a conversion factor [15]. The perceived teeth widths were then multiplied by the conversion factor, to overcome magnification error and achieve the true width captured in the photograph.

### 2.8. Statistical Analysis

The data were analyzed with Statistical Package for the Social Sciences Software (IBM, SPSS Statistics, version 25, Chicago, Illinois, United States). The distribution of data was analysed with normality plots and testing (Shapiro-Wilk and Kolmogorov-Smirnov). Descriptive analysis of categorical (gender) and continuous (age, teeth width, height, and ratios) variables was performed, to calculate frequency, percentage, mean, and standard deviation. Moreover, mean values of dependent variables (width and height ratios of maxillary anterior teeth) were compared using paired *t*-test to detect the mean differences and side disparity. A *p* value ≤ 0.05 was taken as statistically significant.

## 3. Results

The dropout rate of participants in this study was 0.08%. The mean age of the participants was 24.210 ± 3.541. There were 112 (48.7%) male and 118 (51.3%) female participants in this study.

The mean width of maxillary anterior teeth obtained through 2D dental images was recorded 16.114 ± 2.366 for right central incisor, 13.888 ± 5.156 for right lateral incisors, and 11.079 ± 3.093 for a canine tooth. The mean width of the left central incisor was 16.366 ± 5.655, 13.308 ± 1318 lateral incisor, and 10.937 ± 0.803 for a canine tooth.

The mean width of maxillary anterior teeth obtained through 3D dental images was 8.397 ± 0.540 in the right central incisor, 7.735 ± 0.554 right lateral incisor, and 8.042 ± 0.390 for a canine tooth. The mean width of the left central incisor was 8.788 ± 0.426, 7.847 ± 0.620 lateral incisors, and 8.157 ± 0.464 in the canine tooth.

The mean width of maxillary anterior teeth obtained through plaster dental cast was 8.627 ± 0.453 in the right central incisor, 7.371 ± 0.539 in the right lateral incisor, and 7.864 ± 0.457 for a canine tooth. The mean width of the left central incisor was 8.723 ± 0.479, while 7.623 ± 0.637 in the lateral incisor and 7.959 ± 0.482 in the canine tooth (Table 1).

The clean width of maxillary anterior teeth in this study was 8.130 ± 0717 in the right central incisor, while for the lateral incisor, it was 6.241 ± 0.903, and 6.619 ± 1.319 in the canine tooth, whereas in left central incisor 7.965 ± 0.848, lateral incisor 5.983 ± 0.937, and 6.384 ± 1.320 in the canine tooth. There was a significant difference (*p* < 0.05) between the mean values of photographic and clean widths of maxillary anterior teeth (Table 2).

The combined width of maxillary anterior teeth analyzed through 2D photographs were 81.722 ± 9.924, 3D digital models were 48.969 ± 1.508, clean mesiodistal width was 40.788 ± 4.090, and plaster dental cast was 48.170 ± 1.551 (Table 3).

The mean mesiodistal widths of the right central incisor at incisal one-third of the crown was 8.912 ± 0.476, middle third 8.623 ± 0.444, and cervical third 8.354 ± 0.487. The lateral incisor incisal one-third width was 7.826 ± 0.602, the middle third width was 8.623 ± 0.444, and the width at cervical third was 8.354 ± 0.487. Canine tooth incisal one-third width was 8.364 ± 0.457, middle one-third width was 7.864 ± 0.457, while the mean cervical third width was 6.864 ± 0.457.

Similarly, on the left side of the arch, the mean incisal one-third width of the central incisor tooth was 9.219 ± 0.506, middle third width recorded 8.723 ± 0.479, and cervical third 7.765 ± 0.526; as far as the lateral incisor is concerned, the mean incisal one-third width was 8.123 ± 0.637, middle one-third 7.623 ± 0.637, and cervical one-third was 6.623 ± 0.637. Whereas the mean width of the canine tooth at incisal one-third was 8.455 ± 0.475, the middle third was 7.959 ± 0.482, and at the cervical one-third, it was 6.984 ± 0.489 (Table 4).

Furthermore, the mean length of maxillary anterior teeth at different crown levels was the following. The mesial one-third length of the right central incisor was 8.023 ± 0.908, the middle third length was 9.990 ± 0.883, and the distal third length was 7.059 ± 0.921, whereas in lateral incisor, mesial third length was 7.611 ± 0.603, middle third length was 9.093 ± 0.642, while the distal third length was recorded as 6.608 ± 0.613. The mesial third length of the canine tooth was 7.305 ± 0.638, the middle third length was 8.805 ± 0.638, and the cervical third was 7.235 ± 0.635.

However, on the left side of the arch, the mean mesial one-third length of the central incisor tooth was 7.435 ± 0.558, middle third length 8.929 ± 0.520, and the distal third was 7.059 ± 0.623. As far as the lateral incisor is concerned, the mean mesial one-third length was 7.196 ± 0.670, middle one-third length 8.485 ± 0.691, and distal one-third was 7.456 ± 0.713, whereas the mean length of the canine tooth at mesial one-third was 7.281 ± 0.665, middle third 8.839 ± 0.884, and at the distal one-third, it was 6.766 ± 0.775 (Table 5).

The *W*/*H* ratios of maxillary anterior teeth at different crown levels revealed a value of 112.245 ± 13.443 at incisal one-third in the right central incisor, while 87.271 ± 8.798 at the middle third of the crown, and 120.286 ± 16.753 at a cervical third of the crown. Similarly, the width to height ratio at incisal one-third of the right lateral incisor was 99.738 ± 13.479, the middle one-third ratio was 98.965 ± 14.596, whereas at the cervical third, the ratio obtained was 97.143 ± 13.315. The incisal one-third width and height ratio of the right canine was 113.773 ± 17.642, the middle third ratio was 88.651 ± 12.509, and the cervical third was 95.682 ± 14.268.

Moreover, the width to height ratio of maxillary anterior teeth at the left side of the arch revealed a ratio of 124.178 ± 10.436 at incisal one-third in the left central incisor, 98.202 ± 6.781 at the middle third of the crown, while 110.570 ± 9.846 for the cervical third of the crown. Additionally, the width to height ratio at incisal one-third of left lateral incisor was 113.226 ± 15.983, the middle one-third ratio was 89.830 ± 11.437, whereas at the cervical third, the ratio of 89.527 ± 12.697 was found. The incisal one-third width and height ratio of the left canine was 114.978 ± 17.024, the middle third was 96.446 ± 111.824, and the cervical third was 102.848 ± 15.729 (Table 6).

Table 7 is presenting paired *t*-test analysis of crown *W*/*H* ratios at different crown levels. There was a significant difference (*p* = 0.001) between width and height ratio of central incisor at incisal third, supported by a greater *t*-value of -11.932 indicating a large difference between the mean values. The width-height ratio at the middle third was also significant (*p* = 0.001), shown by a large *t*-value (-16.034). Similarly, the cervical third width and height ratios were also statistically significant (*p* = 0.001), and a large *t*-value was noted (7.895).

Furthermore, the comparison of side disparity in maxillary anterior teeth revealed a significant difference (*p* = 0.001) between the width and height ratio of lateral incisor at incisal third, aided by a greater *t*-value of (12.033) indicating a large difference between the mean values. The width-height ratio at the middle third showed a significant difference (*p* = 0.001), supported by a large *t*-value (9.568). Likewise, the cervical third width and height ratio was also statistically significant (*p* = 0.001), endorsed by a large *t*-value (8.092).

However, no significant difference (*p* = 0.296) was found between the width and height ratio of the canine tooth at incisal third level, supported by a small *t*-value of -1.048 indicating no variation between the mean values. The width-height ratio at the middle third was also statistically significant (*p* = 0.001); this finding was supported by a *t*-value (-3.404). Likewise, the cervical third level width and height ratios showed a significant difference (*p* = 0.001) in mean values, indicated by a large *t*-value (-17.145).

## 4. Discussion

The present study is aimed at evaluating the crown width-height ratio of maxillary anterior teeth at different clinical crown levels utilizing 2D photographs and 3D digital dental models. The mean combined mesiodistal widths of maxillary anterior teeth were 81.722 ± 9.924 for 2D photographs, 48.969 ± 1.508 using 3D digital models, 40.788 ± 4.090 clean width after error correction for 2D photographs, and 48.170 ± 1.551 using plaster models. A statistically significant difference (*p* < 0.05) between the mean values of photographic and clean widths of maxillary anterior teeth was seen. The values of photographic width were drastically different form clean width of anterior teeth; the reason is lack of minimizing the photographic error during capture. The images get distorted and enlarged due to difference in a camera focal length, shutter speed, and macro settings; hence, it effects a photograph accuracy and reproducibility. The current study overcomes the distortion effect by adopting the “conversion factor” method proposed by Ward [15] to obtain an accurate and reproducible dental images. The use of such photographic error assessment techniques was also recommended in other studies carried out by Kois [16] and Pitel et al. [17].

In this study, a statistically significant difference (*p* = 0.001) was observed between the width and height ratio of all right and left side anterior teeth at incisal, middle, and cervical thirds except for maxillary canine where the width-height ratio at incisal third was not statistically significant (*p* = 0.296). The crown width and height ratios were measured at different anatomical levels in this study; therefore, a single value of *W*/*H* ratio (73 to 95%) like proposed by Shahid et al. [11] in both sexes that was concluded, based on the middle third width and height of the teeth, and further compared with arch form, arch perimetry and width cannot be compared with our study due to difference in methodology.

In the present study, for maxillary anterior teeth, the greatest crown dimensions were observed for maxillary central incisors, followed by maxillary canines and maxillary lateral incisors. This is by the findings of Alqahtani et al. [2], Sitthiphan et al. [6], Sah et al. [18], Orozco-Varo et al. [19], and Aldegheishem et al. [20]. In the current study, the mean width-height ratio for the right central incisor at the incisal, middle, and cervical thirds of the crown was 112.24%, 87.27%, and 120.286%, respectively (mean: 106.59%), while for the left central incisor, these ratios were 124.17%, 98.2%, and 110.57%, respectively (mean: 110.98%). Likewise, for the right lateral incisor, mean width-height ratios were 99.7%, 98.9%, and 97.1% (mean: 98.56%), and for the left lateral incisor, mean width-height ratios were 113.22%, 89.8%, and 89.52% (mean: 97.5%). For the right canine, mean width-height ratios were 113.77%, 88.65%, and 95.68% (mean: 99.36%), while for the left canine, ratios were 114.97%, 96.44%, and 102.84% (mean: 104.75%). Song et al. [21] in their study carried out similar work but measured the crown width-height ratios at two levels only, namely, mesiodistal width to length (MDW/L) ratio and the cervical width to length (CW/L) ratio. They reported a mean MDW/L of 86% and CW/L of 73% for the central incisors, 84% MDW/L and 67% CW/L for lateral incisors, and 87% MDW/L and 71% CW/L ratio for maxillary canines in the Korean population indicating an increased length of teeth in the said population. These differences can be attributed to differences in ethnicity that have been suggested to affect tooth dimensions among populations [22].

In the present study, the mean width and height of central incisors were 8.59 mm and 8.08 mm, respectively. For lateral incisors, the mean width and length were 7.32 mm and 7.74 mm, while for canine, respective values were 7.75 mm and 7.71 mm. These findings are comparable to those reported by Melo et al. [23] who implied that the findings are not by the “ideal tooth dimensions.” We failed to find any study that evaluated widths and lengths of maxillary anterior teeth at various crown levels, and hence, it was difficult to draw comparisons.

Attempts have been made to curtail possible sources of error and bias in the present study. The main limitation of the study is perhaps its relatively smaller sample size. Future studies with a larger and more diverse sample may reveal interesting results. To limit human error in data collection, all procedures have been carried out by a single operator, where equipment has been used such as cameras and intraoral scanners; the inherent margin of error of the said equipment may have been incorporated, but as such, it does not appear to affect the study results.

In the context of external validity, the results of the present study are generalizable to the Pakistani population in particular and the Southeast Asian population in general. The subjects for this study have been selected from the general population presenting to institute's OPD. Since it is a purely observational study, the effect of any intended interventions and their subsequent outcomes is ruled out by default. If the study were to be repeated in the same population with a different sample, it should yield similar results. However, if subjects from different ethnic backgrounds are selected, results may vary.

## 5. Conclusions

The results of this revealed the following outcome.

1. There was a significant difference when the mean mesiodistal widths and incisocervical lengths of maxillary anterior teeth obtained through 2D photographs were compared with 3D models and standard plaster models, whereas no difference was found between maxillary anterior teeth width of 3D and plaster models. The 3D dental model analysis is accurate and reliable
2. The width and height ratios in the studied population were different at various crown levels. The dimensions of teeth varied from the incisal to the cervical part of the crown. In the current study, the width-height ratio for the right central incisor was 112.24% at incisal, 87.27% middle, and 120.286% at the cervical level of the crown, respectively. The mean *W*/*H* ratio was 106.59%. In the left central incisor, these ratios were 124.17%, 98.2%, and 110.57%, respectively, with a mean ratio of 110.98%
3. Likewise, for the right lateral incisor, width-height ratios were 99.7%, 98.9%, and 97.1%, while the mean ratio was 98.56%. In the left lateral incisor, mean width-height ratios were 113.22%, 89.8%, and 89.52% whereas, the mean ratio was 97.5%
4. The width-height ratios in right canine tooth were 113.77%, 88.65%, and 95.68% (mean: 99.36%), while for the left canine, the ratios were 114.97%, 96.44%, and 102.84% (mean: 104.75%)
5. Hence, rather than relying on a fixed and single ratio of 78% to 80%, the clinician should adopt different width-height ratios to restore teeth with the optimum esthetic outcome

## Figures and Tables

**Table 1:** Distribution of mean maxillary anterior teeth widths obtained from 2D images and 3D and plaster dental models (*n* = 230).

Maxillary teeth	2D photographic width		3D digital model width		Plaster dental cast width	
	Mean	Standard deviation	Mean	Standard deviation	Mean	Standard deviation
Right central incisor	16.114	2.366	8.397	0.540	8.627	0.453
Right lateral incisor	13.888	5.156	7.735	0.554	7.371	0.539
Right canine	11.079	3.093	8.042	0.390	7.864	0.457
Left central incisor	16.366	5.655	8.788	0.426	8.723	0.479
Left lateral incisor	13.308	1.318	7.847	0.620	7.623	0.637
Left canine	10.937	0.803	8.157	0.464	7.959	0.482
Combine six teeth width	81.722	9.924	48.969	1.508	48.170	1.551

2D: two-dimensional; 3D: three-dimensional.

**Table 2:** Comparison of mean maxillary anterior teeth widths obtained from 2D images and clean width obtained after photographic error assessment (*n* = 230).

Maxillary teeth	2D photographic width		Clean width	
	Mean	Standard deviation	Mean	Standard deviation
Right central incisor	16.114	2.366	8.130	0.717
Right lateral incisor	13.888	5.156	6.241	0.903
Right canine	11.079	3.093	6.619	1.319
Left central incisor	16.366	5.655	7.965	0.848
Left lateral incisor	13.308	1.318	5.983	0.937
Left canine	10.937	0.803	6.384	1.320
Combine six teeth width	81.722	9.924	40.788	4.090

Clean width: mesiodistal teeth dimension obtained after photographic error estimation assessment; 2D: two-dimensional (*p* < 0.05).

**Table 3:** Comparison of different combined mesiodistal widths of six maxillary anterior teeth (*n* = 230).

Variables	Mean	Standard deviation
2D photographic	81.722	9.924
3D digital model	48.969	1.508
Clean mesiodistal teeth width	40.788	4.090
Plaster dental cast	48.170	1.551

**Table 4:** Characteristics of mesiodistal widths of maxillary anterior teeth different crown level (*n* = 230).

Variables	Incisal third width	Middle third width	Cervical third width
Right central incisor	8.912 ± 0.476	8.623 ± 0.444	8.354 ± 0.487
Right lateral incisor	7.826 ± 0.602	7.371 ± 0.539	6.366 ± 0.543
Right canine	8.364 ± 0.457	7.864 ± 0.457	6.864 ± 0.457
Left central incisor	9.219 ± 0.506	8.723 ± 0.479	7.765 ± 0.526
Left lateral incisor	8.123 ± 0.637	7.623 ± 0.637	6.623 ± 0.637
Left canine	8.455 ± 0.475	7.959 ± 0.482	6.984 ± 0.489

**Table 5:** Characteristics of incisocervical length of maxillary anterior teeth at different crown levels (*n* = 230).

Variables	Mesial third length	Middle third length	Distal third length
Right central incisor	8.023 ± 0.908	9.990 ± 0.883	7.059 ± 0.921
Right lateral incisor	7.611 ± 0.603	9.093 ± 0.642	6.608 ± 0.613
Right canine	7.305 ± 0.638	8.805 ± 0.638	7.235 ± 0.635
Left central incisor	7.435 ± 0.558	8.929 ± 0.520	7.059 ± 0.623
Left lateral incisor	7.196 ± 0.670	8.485 ± 0.691	7.456 ± 0.713
Left canine	7.281 ± 0.665	8.839 ± 0.884	6.766 ± 0.775

**Table 6:** Distribution of width-height ratios of maxillary anterior teeth at different crown levels (*n* = 230).

Variables	Incisal third width and height ratio % SD	Middle third width and height ratio % SD	Cervical third width and height ratio % SD
Right central incisor	112.245 ± 13.443	87.271 ± 8.798	120.286 ± 16.753
Right lateral incisor	99.738 ± 13.479	98.965 ± 14.596	97.143 ± 13.315
Right canine	113.773 ± 17.642	88.651 ± 12.509	95.682 ± 14.268
Left central incisor	124.178 ± 10.436	98.202 ± 6.781	110.570 ± 9.846
Left lateral incisor	113.226 ± 15.983	89.830 ± 11.437	89.527 ± 12.697
Left canine	114.978 ± 17.024	96.446 ± 111.824	102.848 ± 15.729

%: percentage; SD: standard deviation.

**Table 7:** Comparison of mean width height *W*/*H* ratios of maxillary anterior teeth at different crown levels, paired *T*-test analysis (*n* = 230).

Variables	Incisal third width and height ratio % SD	*p* value	Middle third width and height ratio % SD	*p* value	Cervical third width and height ratio % SD	*p* value
Right central incisor	112.245 ± 13.443	0.001^∗^	87.271 ± 8.798	0.001^∗^	120.286 ± 16.753	0.001^∗^
Left central incisor	124.178 ± 10.436		98.202 ± 6.781		110.570 ± 9.846	
Right lateral incisor	99.738 ± 13.479	0.001^∗^	98.965 ± 14.596	0.001^∗^	97.143 ± 13.315	0.001^∗^
Left lateral incisor	113.226 ± 15.983		89.830 ± 11.437		89.527 ± 12.697	
Right canine	113.773 ± 17.642	0.296	88.651 ± 12.509	0.001^∗^	95.682 ± 14.268	0.001^∗^
Left canine	114.978 ± 17.024		96.446 ± 111.824		102.848 ± 15.729	

^∗^ *p* value less than 0.001; *t*-value: it measures the size of the difference relative to the variation in sample data. The smaller the *t*-value, the more similarity exists between the two sample sets, while a large *t*-score indicates that the groups are different. SD: standard deviation.

## Data Availability

The raw data used to support the findings of this study are included within the article.
